# Protective to a T: The Role of T Cells during Zika Virus Infection

**DOI:** 10.3390/cells8080820

**Published:** 2019-08-03

**Authors:** Ryan D. Pardy, Martin J. Richer

**Affiliations:** 1 Department of Microbiology & Immunology, McGill University, Montreal, QC H3A 2B4, Canada; 2 Rosalind & Morris Goodman Cancer Research Centre, McGill University, Montreal, QC H3G 1Y6, Canada

**Keywords:** zika virus, flaviviruses, T cells, host-pathogen interactions

## Abstract

CD4 and CD8 T cells are an important part of the host’s capacity to defend itself against viral infections. During *flavivirus* infections, T cells have been implicated in both protective and pathogenic responses. Given the recent emergence of Zika virus (ZIKV) as a prominent global health threat, the question remains as to how T cells contribute to anti-ZIKV immunity. Furthermore, high homology between ZIKV and other, co-circulating *flaviviruses* opens the possibility of positive or negative effects of cross-reactivity due to pre-existing immunity. In this review, we will discuss the CD4 and CD8 T cell responses to ZIKV, and the lessons we have learned from both mouse and human infections. In addition, we will consider the possibility of whether T cells, in the context of *flavivirus*-naïve and *flavivirus*-immune subjects, play a role in promoting ZIKV pathogenesis during infection.

## 1. Introduction

T cell responses represent a crucial aspect of the adaptive immune response to infection. In the context of viral infections, both CD4 and CD8 T cells play important roles in controlling and clearing the pathogen. CD4 T cells (or helper T cells) support the immune response through the production of effector cytokines such as interferon (IFN)-γ and tumor necrosis factor (TNF)-α, licensing dendritic cells (DCs) to promote activation of CD8 T cell responses, and activating humoral immunity [1,2]. Meanwhile, CD8 T cells (or cytotoxic T cells) are capable of directly killing infected cells in addition to producing effector cytokines, which makes them critical for controlling viral infections [3]. In addition, both T cell pools are capable of generating long-lived memory populations in order to rapidly respond to re-infection and provide greater protection [3]. While T cell responses during *flavivirus* infections have been shown to be protective, they have also been implicated in pathogenic responses [4]. For example, in mice lacking B cells, CD8 T cells were shown to be critical for controlling yellow fever virus (YFV) infection [5]. In contrast, CD8 T cell infiltration has been associated with increased tissue damage and neurological symptoms in mouse models of Japanese encephalitis virus (JEV) and West Nile virus (WNV) infection [6]. This illustrates that, as is often the case for T cells, T cell responses to *flavivirus* infections must strike a balance between viral control and immunopathology.

Although it was first isolated in 1947, significant research into Zika virus (ZIKV) only began relatively recently [7]. This is primarily due to the fact that it caused only a handful of isolated infections, inducing a mild febrile illness, from its initial isolation until the 21st century [8,9]. However, a series of recent outbreaks in Yap Island, Federated States of Micronesia (2007); French Polynesia (2013); South and Central America (with other outbreaks world-wide; 2015–2016); and India (2018) have demonstrated a novel epidemic capacity for ZIKV [10,11,12,13,14]. Even more striking were novel neurological symptoms associated with ZIKV infection, particularly following the French Polynesian and South and Central American outbreaks [8]. ZIKV has been identified as a potential trigger for Guillain-Barré syndrome (GBS), an autoimmune ascending paralysis that sometimes follows infection [8,15]. However, the most dramatic symptom now associated with ZIKV infection is fetal microcephaly, a neurodevelopmental defect that can cause lifelong complications for newborns [8]. These symptoms—and the outbreaks they were a part—represent a striking change in phenotype for a virus that caused only mild symptoms in its initial characterizations [16,17].

In response to these recent outbreaks and the novel neurological symptoms associated with infection, there has been significant progress in improving our understanding of T cell responses to ZIKV. Broad characterizations of T cell responses induced by ZIKV in humans and mice, including the epitopes of the virus to which they respond, have helped demonstrate protective roles for T cells. These studies have been complemented by cases in which T cell responses have pathogenic consequences for the host. Finally, given the similarities between ZIKV and Dengue virus (DENV), a number of studies have compared T cell responses directed against these two *flaviviruses* to determine whether they are cross-protective or pathogenic. In this review, we will summarize the current understanding of T cell responses during ZIKV infection and the models used to investigate these responses.

## 2. Profiling the T Cell Response to ZIKV Infection

### 2.1. T Cell Responses in Mice

A variety of mouse models have been used to interrogate T cell responses to ZIKV infection. Initially, most models used immunocompromised mice, which typically involved genetic deletion of the IFN-α/β receptor (IFNAR) either globally or in a subset of myeloid cells (LysMCre^+^IFNAR^fl/fl^ mice), or treating with an anti-IFNAR blocking antibody prior to infection [18,19,20,21,22]. The primary lesson from these models is the importance of type I IFN signaling in anti-ZIKV immunity. However, it is also important to consider the impact of IFN deficiency in the context of studying T cell responses to ZIKV. Type I IFNs play a crucial role in promoting the activation of both CD4 and CD8 T cells and are particularly important for enhancing CD8 T cell accumulation and antigen sensitivity [23,24,25,26]. Thus, immunocompetent mouse models represent a very useful tool for characterizing and understanding the CD4 and CD8 T cell responses to ZIKV infection. 

**Figure 1:**
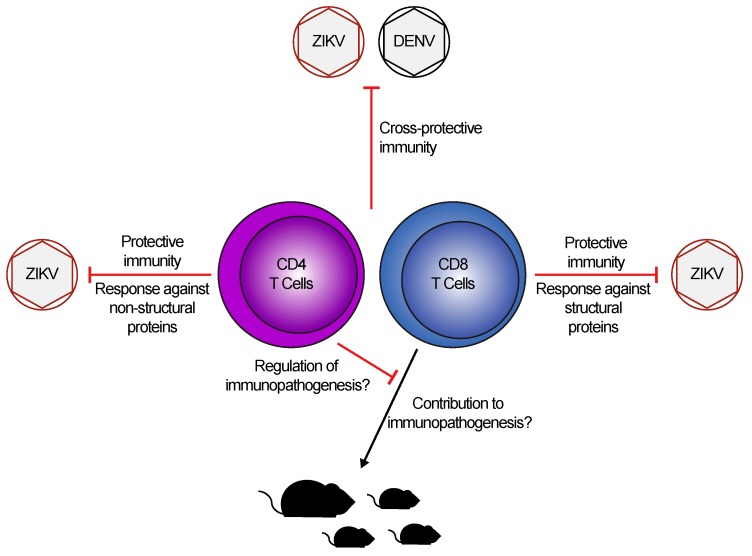
Zika virus (ZIKV) infection induces robust, cross-protective T cell immunity. In both humans and mice, ZIKV infection leads to the generation of Th1 CD4 T cell and effector CD8 T cell responses, which preferentially target epitopes in non-structural and structural proteins, respectively. Studies have shown that immunity to ZIKV is cross-protective against subsequent Dengue virus (DENV) challenge, and vice-versa. Although studies suggest CD8 T cells may contribute to immunopathogenesis in neonatal and adult mice, with CD4 T cells playing a potential regulatory role, this remains to be determined during human infection.

Our group and others have demonstrated that, in immunocompetent mice, ZIKV establishes a self-limiting infection with transient mild weight loss as the only discernible symptom of infection [27,28]. However, infection induces a robust Th1 CD4 T cell response, which features expression of the transcription factor T-bet and production of effector cytokines IFN-γ, TNF-α, and interleukin (IL)-2 [27]. Furthermore, CD8 T cells upregulate expression of IFN-γ and TNF-α, produce the cytolytic molecule granzyme B, and present a highly activated phenotype following ZIKV infection [27,28]. Expansion of this antigen-experienced CD8 T cell population correlated with increased transcripts of type I IFNs [27]. No activation of CD4 or CD8 T cell responses was observed when mice were immunized with UV-inactivated virus, indicating that active infection with live ZIKV is required for the generation of CD4 and CD8 T cell-mediated immunity [27]. These models were used to identify an immunodominant CD8 T cell epitope in the ZIKV envelope protein, highlighting the specificity of the approaches used to quantify and characterize the T cell responses to ZIKV infection (Figure 1) [27,28]. An additional study using intracranial infection of immunocompetent mice described a functional role for T cells during ZIKV infection. When mice were infected intravenously, followed by intracranial infection four weeks later, they were protected from the high central nervous system (CNS) viral load and severe disease observed in mice that were only infected intracranially [29]. However, this protection was lost in T cell-deficient mice, demonstrating a key role for T cells in controlling intracranial ZIKV infection and pathology [29]. Together, these studies demonstrate that ZIKV actively infects immunocompetent mice, generating a robust and functional CD4 and CD8 T cell response to infection. 

Similar findings have been observed in LysMCre^+^IFNAR^fl/fl^ mice, which lack IFNAR in mature macrophages and granulocytes, with a partial deletion in CD11c^+^ splenic dendritic cells [18,30,31]. In this model, ZIKV infection caused an increased frequency of activated CD8 T cells in the spleen, which were shown to be positive for granzyme B [18]. This model was used to identify several CD8 T cell epitopes, including the aforementioned immunodominant epitope in the ZIKV envelope protein [18,27,28]. When CD8 T cells were depleted in this model, mice had higher viral burdens in the serum, CNS, and other tissues. This was reversed when CD8 T cell-depleted mice received a transfer of memory CD8 T cells [18]. Further studies in LysMCre^+^IFNAR^fl/fl^ mice have described a similar Th1 CD4 T cell response to what was observed in immunocompetent mice, as well as a T follicular helper (Tfh) cell response from 7 days post-infection (dpi) onward [19]. CD4 T cells were required for the generation of an immunoglobulin G (IgG) antibody response, but their depletion had no impact on the CD8 T cell response, and nor did it impact viral burden [19]. Thus, although IFNAR expression on certain myeloid cells is not strictly required for the generation of T cell responses, the impact its deletion has on T cell accumulation is unclear as only frequencies were reported in these studies. Similarly, it is unknown whether CD8 T cells in LysMCre^+^IFNAR^fl/fl^ mice are functionally impaired since analyses of CD8 T cell capacity to produce IFN-γ, killing capacity, or antigen sensitivity were either not undertaken or not compared to wild-type (WT) mice [18]. It also warrants further investigation into whether there was an impact on the generation of antigen-specific T cell responses since only the immunodominant CD8 T cell epitope in the ZIKV envelope protein is shared between this study and other studies [18,27,28].

Although IFNAR deficient mice are known to be susceptible to ZIKV infection [32,33,34], depleting CD4 T cells from 10–12 week old IFNAR knock-out (KO) mice caused higher viral loads, more severe paralysis, and reduced survival [20], and caused lethal infection in 3-4 week old IFNAR KO mice [21]. Each of these studies found that transferring memory CD4 T cells from ZIKV-immune mice, but not naïve mice, was protective against a subsequent lethal ZIKV challenge [20,21]. Lucas et al. found this protection to be dependent on IFN-γ signaling and B cells in the recipient mice, which suggests that CD4 T cells were important for promoting B cell and antibody responses against ZIKV (Figure 1) [21]. Similarly, ZIKV infection in CD8 T cell depleted, IFNAR-deficient mice was lethal, as was infection of IFNAR-blocking antibody-treated *Rag1^−/−^* mice (which lack both T and B cells) [21,35]. In the latter case, WT control mice treated with the IFNAR blocking antibody do not succumb to infection [35], demonstrating the importance of adaptive immunity in protection from lethal ZIKV infection. Finally, antibody-mediated blocking of IFNAR, followed by intravaginal infection, enables the virus to spread systemically despite increased frequency of tetramer-specific CD8 T cells and total CD4 T cells in the lower female reproductive tract [22]. Depleting both CD4 and CD8 T cells together, but not individually, led to a loss of viral control, suggesting that each subset is able to compensate for the loss of the other [22]. Thus, even in IFN-deficient mouse models, CD4 and CD8 T cells continue to play an important role in the immune response to ZIKV. 

In all, these studies demonstrate the importance of robust CD4 and CD8 T cell responses during ZIKV infection. Broad characterizations of the T cell response in immunocompetent mice will serve as an important baseline, to which the T cell response to contemporary ZIKV isolates may be compared. Differences in T cell responses induced by epidemic strains of ZIKV could improve our understanding of how the virus has changed and whether this has had an impact on its pathogenesis. Although the studies in immunocompromised mice have highlighted the importance of T cell responses in these circumstances, human infection with ZIKV is rarely, if ever, fatal. Therefore, the findings from these models must be analyzed under the prism that such severe phenotypes are rarely observed during the course of natural infections in humans.

### 2.2. T Cell Responses in Humans

One focus of the limited number of studies characterizing human T cell responses to ZIKV has been to identify immunogenic epitopes and their locations within the ZIKV proteome. In a cohort of 45 American patients with confirmed ZIKV infection, highly polyfunctional CD4 and CD8 T cells responses were detected following stimulation with pools of 15mer peptides (overlapping by 11 peptides) from all ZIKV proteins [36]. They found that although 89% of patients’ CD4 T cells responded to peptides from the capsid and envelope proteins, the most robust IFN-γ production was following stimulation with peptides from the non-structural (NS)1, NS3, and NS5 proteins [36]. Conversely, CD8 T cell responses against the NS3, NS4B, and NS5 proteins were detected in most patients, but the most robust IFN-γ responses were against the capsid and envelope proteins [36]. Similarly, a case report from Florida identified NS2-specific CD4 T cells and envelope-specific CD8 T cells in a returning traveler with ZIKV infection [37]. A case series featuring five returning American travelers with ZIKV infection identified very modest, but detectable CD4 and CD8 T cell responses (<1% cytokine-producing among total CD4 or CD8 T cells) against pooled peptides from the capsid, pre-membrane, envelope, and NS5 proteins, although no other proteins were tested [38]. Two additional case reports from the same group found consistent CD4 T cell responses against NS1, NS3, and NS5 proteins, and CD8 T cell responses against envelope, NS3, and NS5 proteins [39,40]. In all, a common theme of these characterizations is a tendency for CD8 T cells to respond to structural proteins (primarily capsid and envelope proteins), and for CD4 T cells to respond to NS proteins (mainly NS1, NS3, and NS5 proteins; Figure 1). It is interesting to note that these results are reflective of the results found in mouse studies, although it is unlikely that the epitope peptide sequences would be the same [18,19,27,28]. 

The other question often addressed by human studies relates to how T cell responses to ZIKV change over time. In the case report from Florida described above, T cell responses appeared 7 days post-onset of symptoms (POS), peaked 21 days POS, and memory T cell responses were detectable as late as 148 days POS [37]. Similarly, the T cell responses described in the 45-patient American cohort were tracked into memory time points as late as 10 to 12 months POS [36]. One study took an alternative approach, tracking cytokine responses and cellular dynamics in the blood over time [41]. In this cohort of 55 Singaporean patients, viremic patients in the acute phase of infection had reduced numbers of immune cells in their blood (including CD4 and CD8 T cells), and higher production of IFN-γ [41]. T cell cytokines IFN-γ and IL-12, and chemokine CCL5 (also known as Regulated upon Activation, Normal T cell Expressed, and Secreted, or RANTES) were maintained into the convalescent phase of infection (10–35 days POS) [41]. Interestingly, the authors found significantly more CD4 and CD8 T cells in the blood of non-viremic patients with moderate symptoms when compared to viremic patients with moderate symptoms, suggesting a possible involvement of T cell responses in the clearance of viremia [41]. Finally, in an Italian cohort, ZIKV infection activated both CD4 and CD8 T cells, but only CD4 T cells acquired an effector memory phenotype when compared to CD4 T cells from healthy controls [42]. This study also identified an increase in granzyme B-producing, double-negative T cells, which expressed the Vδ2 T cell receptor [42]. The authors highlight this observation because Vδ2 T cells have been implicated in recurrent abortions, although they have never been associated with ZIKV-induced fetal complications [42]. The overarching trend, however, is for ZIKV to induce long-lasting CD4 and CD8 T cell responses which span both structural and NS proteins (Figure 1).

## 3. T Cell Responses to DENV and ZIKV: Cross-Protective or Pathogenic?

Given the similarity between DENV and ZIKV and their shared regions of endemicity, obvious questions have emerged as to whether immunity to one virus can cross-protect against the other. However, immune responses against distinct DENV serotypes have also been suggested to worsen disease outcomes through distinct B- and T-cell dependent mechanisms. In the context of B cells, antibody-dependent enhancement (ADE) occurs when sub-neutralizing antibody responses generated during a primary infection recognize and bind to cross-reactive epitopes from the secondary, heterologous infection. When the virus particles and sub-neutralizing antibodies are subsequently internalized via Fc receptor-mediated endocytosis, the virus remains capable of replicating within the cell [43]. ADE has been associated with reduced antiviral responses and may enable DENV to infect cells that are normally non-permissive to infection [43]. With T cells, original antigen sin (OAS) occurs when a heterologous secondary infection activates memory T cells that recognize similar but distinct antigens that were present during the primary infection. The result is that these memory T cells mount an ineffective response against the secondary infection, which prevents a more effective T cell response from being generated [44]. As such, significant research has focused on the impact of prior DENV immunity on the immune response to ZIKV or the impact of ZIKV immunity on the immune response to DENV infection.

The majority of human studies of cross-reactivity have investigated the ability of DENV- or ZIKV-derived peptides to restimulate T cells from the heterologous infection. As a whole, these papers consistently identify cross-reactivity in both CD4 and CD8 T cell responses, targeting a variety of viral proteins (Figure 1) [45,46,47,48,49]. In particular, one study identified capsid and envelope protein-specific CD4 T cell responses following ZIKV infection [46]. Upon comparing the peptide sequences of the epitopes to previously identified epitopes in the capsid and envelope proteins from YFV, DENV, and tick-borne encephalitis virus (TBEV), they found that, while the epitopes were all located in similar regions of the proteins, surprisingly they did not share similar sequence identity. As such, this suggests that sequence identity was not the driving factor in the conservation of these epitopes [46]. Functionally, studies have also shown that prior DENV immunity has no impact on the ability of CD4 or CD8 T cells to produce IFN-γ or TNF-α [50]. However, patients co-infected with both DENV and ZIKV had a slight decrease in the frequency of IFN-γ^+^ or TNF-α^+^ CD4 T cells and similar frequencies of IFN-γ^+^ or TNF-α^+^ CD8 T cells compared to patients infected with DENV or ZIKV alone, although the implications of this finding remain unclear [50]. Another group found that while prior DENV exposure had no impact on the CD4 T cell response to ZIKV infection, patients in the acute phase of ZIKV infection had more IFN-γ^+^ CD8 T cells after restimulation with ZIKV-derived peptides [47]. Further, the CD8 T cell response to ZIKV in DENV-naïve patients was more targeted to structural proteins, while DENV-immune patients’ responses were shifted toward the NS proteins [47]. However, prior DENV immunity had no impact on either the transcriptional profile of the CD8 T cells, nor the capacity of CD4 or CD8 T cells to produce IFN-γ at later stages of infection [47,51]. Together, these studies demonstrate a high degree of cross-reactivity between T cell responses to DENV and ZIKV and, thus far, have given no indication that this may have a negative impact on disease outcomes.

Mouse models have been particularly useful for determining the impact of prior DENV or ZIKV immunity on infection outcomes and disease severity. For example, transgenic mice expressing human leukocyte antigen (HLA) class I were crossed with IFNAR KO mice to identify ZIKV-derived peptides that could contribute to human CD8 T cell responses [52]. Several of these peptides were found to restimulate CD8 T cells from DENV-infected mice, and immunization with the ZIKV/DENV cross-reactive peptides induced a CD8 T cell response that reduced ZIKV viral loads in the serum and brain [52]. Similarly, HLA class II transgenic mice were used to identify several potential CD4 T cell epitopes, many of which had homologous and cross-reactive sequences in DENV, WNV, and YFV [53]. Further evidence of a cross-protective role for CD8 T cells was described by transferring memory CD8 T cells isolated from DENV-immune IFNAR KO mice to naïve IFNAR KO mice, which provided protection from ZIKV-induced weight loss, morbidity, and mortality [54]. Finally, one study has analyzed the impact of prior *flavivirus* immunity during pregnancy [55]. They observed that DENV-immune pregnant females had lower ZIKV viral loads in the spleen and placenta and were completely protected from fetal resorption following ZIKV infection [55]. This protection was dependent on the presence of DENV-specific memory CD8 T cells, and their depletion resulted in higher viral loads in maternal tissues and fetal resorption [55]. There is clearly a high degree of cross-reactivity between the T cell responses against ZIKV and related *flaviviruses*, which may play an important role in host protection, particularly in the context of pregnancy. However, the ZIKV outbreak in South and Central America represented the introduction of ZIKV to an area where DENV and other *flaviviruses* already circulate. It is reasonable to assume that a high degree of prior *flavivirus* immunity exists in these populations, yet fetal microcephaly was one of the most striking symptoms associated with this outbreak [8]. It is also worth noting that the above study took place in IFNAR KO dams, raising the question of how the loss of a key part of the antiviral immune system impacts the observed phenotype. Further research is therefore needed to reconcile the protection from fetal pathology observed in DENV-immune dams with the increase in fetal microcephaly observed during the South American outbreak. 

## 4. Role of T Cells in ZIKV Pathogenesis

Given the ability of CD8 T cells to directly kill infected cells, and the capacity of both CD4 and CD8 T cells to produce effector cytokines and cytolytic molecules, it is intriguing to ask whether the T cell response induced by ZIKV could contribute to pathogenesis. Immunopathogenic T cell responses have been described primarily in the context of influenza A virus infection, during which T cell infiltration and TNF-α production promote viral clearance while also contributing to lung pathology [56]. Similarly, Schmidt and colleagues observed exacerbated morbidity and mortality when mice with memory CD8 T cells (but no memory CD4 T cells or antibodies) against respiratory syncytial virus (RSV) were re-challenged with RSV [57]. CD8 T cell responses to TBEV have been linked to reduced survival in mouse studies, and increased cell death in infected human neuronal tissue [58,59]. In the context of ZIKV infection, a pair of studies took the approach of infecting WT neonatal mice (one day old), which caused chorioretinal lesions and neuronal degeneration [60,61]. This tissue-damaging phenotype correlated with CD8 T cell infiltration into the eye and CNS, respectively, suggesting a potential role for CD8 T cells in mediating the damage (Figure 1) [60,61]. These observations are reflective of several studies that have examined ZIKV infection in neonatal mice, which leads to central nervous system infection and pathology, and is often fatal [32,62,63,64]. Although this may provide some insight into pathogenesis in fetuses or infants, neonatal mice do not possess a fully mature immune system and are highly susceptible to infection with neurotropic viruses [65,66]. As such, using this model to analyze immune responses must be undertaken with these caveats in mind. 

Using IFNAR KO mice, Jurado et al. have described a clearer role for CD8 T cells in causing brain damage and paralysis during ZIKV infection [67]. They found significant CD8 T cell infiltration into the brain following ZIKV infection, which correlated with paralysis and death of all mice by 9 dpi [67]. Upon depletion of CD8 T cells, they observed reduced paralysis and improved survival, although mice had higher viral loads in the brain, suggesting that while CD8 T cells are important for viral clearance, they can cause significant immunopathology in the brain (Figure 1) [67]. When both CD4 and CD8 T cells were depleted, mice had an intermediate phenotype with a significant decrease in survival, suggesting a regulatory role for CD4 T cells in the brain (Figure 1) [67]. This potential regulatory role is supported by depletion of CD4 T cells alone, which caused all mice to develop paralysis and succumb to infection [67]. Another group observed that CD4 T cell depletion had no impact on the CD8 T cell response, but caused significantly higher viral loads in the CNS, worsened disease, and decreased survival [20]. Together, these studies implicate CD8 T cells in contributing to ZIKV pathogenesis in immune-privileged sites, while they describe a potential regulatory role for CD4 T cells. 

## 5. Conclusions

Since the beginning of the South American ZIKV outbreak, significant research has been conducted to further our understanding of T cell responses to ZIKV infection. In both humans and mouse models of infection, ZIKV induces robust T cell activation, which leads to the establishment of a memory T cell population, suggesting an important role for CD4 and CD8 T cells in the immune response to ZIKV. This is highlighted by depletion studies, in which loss of either CD4, CD8, or both T cell subsets together can result in worsened morbidity, mortality, or even fetal resorption. Identification of ZIKV epitopes, in particular broadly conserved epitopes between studies, and even among *flaviviruses*, could provide novel candidates for vaccine design. Given that the work done so far studying T cell cross-reactivity has demonstrated a protective role for these cells, it stands to reason that cross-reactive epitopes could be useful in vaccination against multiple, co-circulating *flaviviruses*. However, this remains to be formally tested, and the magnitude of the South and Central American ZIKV outbreak in a DENV endemic region suggests that prior DENV immunity may not provide complete protection. Finally, there may be a role for CD8 T cells in enhancing ZIKV pathogenesis, although thus far studies have been completed uniquely in extremely young or immunocompromised mice, raising questions as to whether CD8 T cells also play a role in ZIKV pathogenesis in healthy, immunocompetent adults. In the future, it will be of importance to continue to explore the impact of prior immunity to *flaviviruses* during pregnancy. Further research is also needed to understand whether ZIKV has improved its capacity to evade host immune responses, including T cell-mediated immunity, and whether this has contributed to the increased pathogenesis observed during recent outbreaks.

## References

[1] Luckheeram R.V., Zhou R., Verma A.D., Xia B. (2012). CD4^+^ T cells: Differentiation and functions. Clin. Dev. Immunol..

[2] Smith C.M., Wilson N.S., Waithman J., Villadangos J.A., Carbone F.R., Heath W.R., Belz G.T. (2004). Cognate CD4^+^ T cell licensing of dendritic cells in CD8^+^ T cell immunity. Nat. Immunol.

[3] Valbon S.F., Condotta S.A., Richer M.J. (2016). Regulation of effector and memory CD8^+^ T cell function by inflammatory cytokines. Cytokine.

[4] Slon Campos J.L., Mongkolsapaya J., Screaton G.R. (2018). The immune response against flaviviruses. Nat. Immunol..

[5] Bassi M.R., Kongsgaard M., Steffensen M.A., Fenger C., Rasmussen M., Skjodt K., Finsen B., Stryhn A., Buus S., Christensen J.P. (2015). CD8^+^ T cells complement antibodies in protecting against yellow fever virus. J. Immunol..

[6] Wang Y., Lobigs M., Lee E., Mullbacher A. (2003). CD8^+^ T cells mediate recovery and immunopathology in West Nile virus encephalitis. J. Virol..

[7] Dick G.W., Kitchen S.F., Haddow A.J. (1952). Zika virus. I. Isolations and serological specificity. Trans. R. Soc. Trop. Med. Hyg..

[8] Rajah M.M., Pardy R.D., Condotta S.A., Richer M.J., Sagan S.M. (2016). Zika Virus: Emergence, Phylogenetics, Challenges, and Opportunities. ACS Infect. Dis..

[9] Kindhauser M.K., Allen T., Frank V., Santhana R.S., Dye C. (2016). Zika: The origin and spread of a mosquito-borne virus. Bull. World Health Organ..

[10] Duffy M.R., Chen T.H., Hancock W.T., Powers A.M., Kool J.L., Lanciotti R.S., Pretrick M., Marfel M., Holzbauer S., Dubray C. (2009). Zika virus outbreak on Yap Island, Federated States of Micronesia. N. Engl. J. Med..

[11] Oehler E., Watrin L., Larre P., Leparc-Goffart I., Lastere S., Valour F., Baudouin L., Mallet H., Musso D., Ghawche F. (2014). Zika virus infection complicated by Guillain-Barre syndrome—Case report, French Polynesia, December 2013. Eurosurveillance.

[12] The History of Zika Virus. http://www.who.int/emergencies/zika-virus/history/en/.

[13] Swati G. (2018). Zika Spreads Rapidly in India, with 94 Cases Confirmed.

[14] Slater J. (2018). India Wrestles with First Significant Outbreak of Zika Virus.

[15] Krauer F., Riesen M., Reveiz L., Oladapo O.T., Martinez-Vega R., Porgo T.V., Haefliger A., Broutet N.J., Low N., Group W.H.O.Z.C.W. (2017). Zika Virus Infection as a Cause of Congenital Brain Abnormalities and Guillain-Barre Syndrome: Systematic Review. PLoS Med..

[16] Dick G.W. (1952). Zika virus. II. Pathogenicity and physical properties. Trans. R Soc. Trop. Med. Hyg..

[17] Simpson D.I. (1964). Zika Virus Infection in Man. Trans. R. Soc. Trop. Med. Hyg..

[18] Elong Ngono A., Vizcarra E.A., Tang W.W., Sheets N., Joo Y., Kim K., Gorman M.J., Diamond M.S., Shresta S. (2017). Mapping and Role of the CD8^+^ T Cell Response During Primary Zika Virus Infection in Mice. Cell Host Microbe.

[19] Elong Ngono A., Young M.P., Bunz M., Xu Z., Hattakam S., Vizcarra E., Regla-Nava J.A., Tang W.W., Yamabhai M., Wen J. (2019). CD4^+^ T cells promote humoral immunity and viral control during Zika virus infection. PLoS Pathog..

[20] Hassert M., Wolf K.J., Schwetye K.E., DiPaolo R.J., Brien J.D., Pinto A.K. (2018). CD4^+^ T cells mediate protection against Zika associated severe disease in a mouse model of infection. PLoS Pathog.

[21] Lucas C.G.O., Kitoko J.Z., Ferreira F.M., Suzart V.G., Papa M.P., Coelho S.V.A., Cavazzoni C.B., Paula-Neto H.A., Olsen P.C., Iwasaki A. (2018). Critical role of CD4^+^ T cells and IFNgamma signaling in antibody-mediated resistance to Zika virus infection. Nat. Commun.

[22] Scott J.M., Lebratti T.J., Richner J.M., Jiang X., Fernandez E., Zhao H., Fremont D.H., Diamond M.S., Shin H. (2018). Cellular and Humoral Immunity Protect against Vaginal Zika Virus Infection in Mice. J. Virol..

[23] Kolumam G.A., Thomas S., Thompson L.J., Sprent J., Murali-Krishna K. (2005). Type I interferons act directly on CD8 T cells to allow clonal expansion and memory formation in response to viral infection. J. Exp. Med..

[24] Curtsinger J.M., Mescher M.F. (2010). Inflammatory cytokines as a third signal for T cell activation. Curr. Opin. Immunol..

[25] Richer M.J., Nolz J.C., Harty J.T. (2013). Pathogen-specific inflammatory milieux tune the antigen sensitivity of CD8^+^ T cells by enhancing T cell receptor signaling. Immunity.

[26] Longhi M.P., Trumpfheller C., Idoyaga J., Caskey M., Matos I., Kluger C., Salazar A.M., Colonna M., Steinman R.M. (2009). Dendritic cells require a systemic type I interferon response to mature and induce CD4^+^ Th1 immunity with poly IC as adjuvant. J. Exp. Med..

[27] Pardy R.D., Rajah M.M., Condotta S.A., Taylor N.G., Sagan S.M., Richer M.J. (2017). Analysis of the T Cell Response to Zika Virus and Identification of a Novel CD8^+^ T Cell Epitope in Immunocompetent Mice. PLoS Pathog..

[28] Huang H., Li S., Zhang Y., Han X., Jia B., Liu H., Liu D., Tan S., Wang Q., Bi Y. (2017). CD8^+^ T Cell Immune Response in Immunocompetent Mice during Zika Virus Infection. J. Virol..

[29] Nazerai L., Scholler A.S., Rasmussen P.O.S., Buus S., Stryhn A., Christensen J.P., Thomsen A.R. (2018). A New In Vivo Model to Study Protective Immunity to Zika Virus Infection in Mice With Intact Type I Interferon Signaling. Front. Immunol..

[30] Clausen B.E., Burkhardt C., Reith W., Renkawitz R., Forster I. (1999). Conditional gene targeting in macrophages and granulocytes using LysMcre mice. Transgenic Res..

[31] Diamond M.S., Kinder M., Matsushita H., Mashayekhi M., Dunn G.P., Archambault J.M., Lee H., Arthur C.D., White J.M., Kalinke U. (2011). Type I interferon is selectively required by dendritic cells for immune rejection of tumors. J. Exp. Med..

[32] Lazear H.M., Govero J., Smith A.M., Platt D.J., Fernandez E., Miner J.J., Diamond M.S. (2016). A Mouse Model of Zika Virus Pathogenesis. Cell Host Microbe.

[33] Rossi S.L., Tesh R.B., Azar S.R., Muruato A.E., Hanley K.A., Auguste A.J., Langsjoen R.M., Paessler S., Vasilakis N., Weaver S.C. (2016). Characterization of a Novel Murine Model to Study Zika Virus. Am. J. Trop Med. Hyg..

[34] Dowall S.D., Graham V.A., Rayner E., Atkinson B., Hall G., Watson R.J., Bosworth A., Bonney L.C., Kitchen S., Hewson R. (2016). A Susceptible Mouse Model for Zika Virus Infection. PLoS Negl. Trop. Dis..

[35] Winkler C.W., Myers L.M., Woods T.A., Messer R.J., Carmody A.B., McNally K.L., Scott D.P., Hasenkrug K.J., Best S.M., Peterson K.E. (2017). Adaptive Immune Responses to Zika Virus Are Important for Controlling Virus Infection and Preventing Infection in Brain and Testes. J. Immunol..

[36] El Sahly H.M., Gorchakov R., Lai L., Natrajan M.S., Patel S.M., Atmar R.L., Keitel W.A., Hoft D.F., Barrett J., Bailey J. (2019). Clinical, Virologic, and Immunologic Characteristics of Zika Virus Infection in a Cohort of US Patients: Prolonged RNA Detection in Whole Blood. Open Forum Infect. Dis..

[37] Ricciardi M.J., Magnani D.M., Grifoni A., Kwon Y.C., Gutman M.J., Grubaugh N.D., Gangavarapu K., Sharkey M., Silveira C.G.T., Bailey V.K. (2017). Ontogeny of the B- and T-cell response in a primary Zika virus infection of a dengue-naive individual during the 2016 outbreak in Miami, FL. PLoS Negl. Trop. Dis..

[38] Lai L., Rouphael N., Xu Y., Natrajan M.S., Beck A., Hart M., Feldhammer M., Feldpausch A., Hill C., Wu H. (2018). Innate, T-, and B-Cell Responses in Acute Human Zika Patients. Clin. Infect. Dis..

[39] Edupuganti S., Natrajan M.S., Rouphael N., Lai L., Xu Y., Feldhammer M., Hill C., Patel S.M., Johnson S.J., Bower M. (2017). Biphasic Zika Illness With Rash and Joint Pain. Open Forum Infect. Dis..

[40] Waggoner J.J., Rouphael N., Xu Y., Natrajan M., Lai L., Patel S.M., Levit R.D., Edupuganti S., Mulligan M.J. (2017). Pericarditis Associated With Acute Zika Virus Infection in a Returning Traveler. Open Forum Infect. Dis..

[41] Lum F.M., Lye D.C.B., Tan J.J.L., Lee B., Chia P.Y., Chua T.K., Amrun S.N., Kam Y.W., Yee W.X., Ling W.P. (2018). Longitudinal Study of Cellular and Systemic Cytokine Signatures to Define the Dynamics of a Balanced Immune Environment During Disease Manifestation in Zika Virus-Infected Patients. J. Infect. Dis..

[42] Cimini E., Castilletti C., Sacchi A., Casetti R., Bordoni V., Romanelli A., Turchi F., Martini F., Tumino N., Nicastri E. (2017). Human Zika infection induces a reduction of IFN-gamma producing CD4 T-cells and a parallel expansion of effector Vdelta2 T-cells. Sci. Rep..

[43] Halstead S.B., Ackerman M.E. (2014). Pathogenic exploitation of Fc activity. Antibody Fc: Linking Adaptive and Innate Immunity.

[44] Klenerman P., Zinkernagel R.M. (1998). Original antigenic sin impairs cytotoxic T lymphocyte responses to viruses bearing variant epitopes. Nature.

[45] Lim M.Q., Kumaran E.A.P., Tan H.C., Lye D.C., Leo Y.S., Ooi E.E., MacAry P.A., Bertoletti A., Rivino L. (2018). Cross-Reactivity and Anti-viral Function of Dengue Capsid and NS3-Specific Memory T Cells Toward Zika Virus. Front. Immunol..

[46] Koblischke M., Stiasny K., Aberle S.W., Malafa S., Tschouchnikas G., Schwaiger J., Kundi M., Heinz F.X., Aberle J.H. (2018). Structural Influence on the Dominance of Virus-Specific CD4 T Cell Epitopes in Zika Virus Infection. Front. Immunol..

[47] Grifoni A., Pham J., Sidney J., O'Rourke P.H., Paul S., Peters B., Martini S.R., de Silva A.D., Ricciardi M.J., Magnani D.M. (2017). Prior Dengue Virus Exposure Shapes T Cell Immunity to Zika Virus in Humans. J. Virol..

[48] Paquin-Proulx D., Leal F.E., Terrassani Silveira C.G., Maestri A., Brockmeyer C., Kitchen S.M., Cabido V.D., Kallas E.G., Nixon D.F. (2017). T-cell Responses in Individuals Infected with Zika Virus and in Those Vaccinated Against Dengue Virus. Pathog. Immun..

[49] Herrera B.B., Tsai W.Y., Brites C., Luz E., Pedroso C., Drexler J.F., Wang W.K., Kanki P.J. (2018). T Cell Responses to Nonstructural Protein 3 Distinguish Infections by Dengue and Zika Viruses. MBio.

[50] Badolato-Correa J., Sanchez-Arcila J.C., Alves de Souza T.M., Santos Barbosa L., Conrado Guerra Nunes P., da Rocha Queiroz Lima M., Gandini M., Bispo de Filippis A.M., Venancio da Cunha R., Leal de Azeredo E. (2018). Human T cell responses to Dengue and Zika virus infection compared to Dengue/Zika coinfection. Immun. Inflamm. Dis..

[51] Grifoni A., Costa-Ramos P., Pham J., Tian Y., Rosales S.L., Seumois G., Sidney J., de Silva A.D., Premkumar L., Collins M.H. (2018). Cutting Edge: Transcriptional Profiling Reveals Multifunctional and Cytotoxic Antiviral Responses of Zika Virus-Specific CD8^+^ T Cells. J. Immunol..

[52] Wen J., Tang W.W., Sheets N., Ellison J., Sette A., Kim K., Shresta S. (2017). Identification of Zika virus epitopes reveals immunodominant and protective roles for dengue virus cross-reactive CD8^+^ T cells. Nat. Microbiol.

[53] Reynolds C.J., Suleyman O.M., Ortega-Prieto A.M., Skelton J.K., Bonnesoeur P., Blohm A., Carregaro V., Silva J.S., James E.A., Maillere B. (2018). T cell immunity to Zika virus targets immunodominant epitopes that show cross-reactivity with other Flaviviruses. Sci. Rep..

[54] Wen J., Elong Ngono A., Regla-Nava J.A., Kim K., Gorman M.J., Diamond M.S., Shresta S. (2017). Dengue virus-reactive CD8^+^ T cells mediate cross-protection against subsequent Zika virus challenge. Nat. Commun..

[55] Regla-Nava J.A., Elong Ngono A., Viramontes K.M., Huynh A.T., Wang Y.T., Nguyen A.T., Salgado R., Mamidi A., Kim K., Diamond M.S. (2018). Cross-reactive Dengue virus-specific CD8^+^ T cells protect against Zika virus during pregnancy. Nat. Commun..

[56] Peiris J.S., Hui K.P., Yen H.L. (2010). Host response to influenza virus: Protection versus immunopathology. Curr. Opin. Immunol..

[57] Schmidt M.E., Knudson C.J., Hartwig S.M., Pewe L.L., Meyerholz D.K., Langlois R.A., Harty J.T., Varga S.M. (2018). Memory CD8 T cells mediate severe immunopathology following respiratory syncytial virus infection. PLoS Pathog..

[58] Gelpi E., Preusser M., Laggner U., Garzuly F., Holzmann H., Heinz F.X., Budka H. (2006). Inflammatory response in human tick-borne encephalitis: Analysis of postmortem brain tissue. J. Neurovirol..

[59] Ruzek D., Salat J., Palus M., Gritsun T.S., Gould E.A., Dykova I., Skallova A., Jelinek J., Kopecky J., Grubhoffer L. (2009). CD8^+^ T-cells mediate immunopathology in tick-borne encephalitis. Virology.

[60] Manangeeswaran M., Ireland D.D., Verthelyi D. (2016). Zika (PRVABC59) Infection Is Associated with T cell Infiltration and Neurodegeneration in CNS of Immunocompetent Neonatal C57Bl/6 Mice. PLoS Pathog.

[61] Manangeeswaran M., Kielczewski J.L., Sen H.N., Xu B.C., Ireland D.D.C., McWilliams I.L., Chan C.C., Caspi R.R., Verthelyi D. (2018). ZIKA virus infection causes persistent chorioretinal lesions. Emerg. Microbes. Infect..

[62] Li S., Armstrong N., Zhao H., Hou W., Liu J., Chen C., Wan J., Wang W., Zhong C., Liu C. (2018). Zika Virus Fatally Infects Wild Type Neonatal Mice and Replicates in Central Nervous System. Viruses.

[63] Miner J.J., Sene A., Richner J.M., Smith A.M., Santeford A., Ban N., Weger-Lucarelli J., Manzella F., Ruckert C., Govero J. (2016). Zika Virus Infection in Mice Causes Panuveitis with Shedding of Virus in Tears. Cell Rep..

[64] Wu Y.H., Tseng C.K., Lin C.K., Wei C.K., Lee J.C., Young K.C. (2018). ICR suckling mouse model of Zika virus infection for disease modeling and drug validation. PLoS Negl. Trop. Dis..

[65] Couderc T., Chretien F., Schilte C., Disson O., Brigitte M., Guivel-Benhassine F., Touret Y., Barau G., Cayet N., Schuffenecker I. (2008). A mouse model for Chikungunya: Young age and inefficient type-I interferon signaling are risk factors for severe disease. PLoS Pathog..

[66] Pedras-Vasconcelos J.A., Puig M., Sauder C., Wolbert C., Ovanesov M., Goucher D., Verthelyi D. (2008). Immunotherapy with CpG oligonucleotides and antibodies to TNF-alpha rescues neonatal mice from lethal arenavirus-induced meningoencephalitis. J. Immunol..

[67] Jurado K.A., Yockey L.J., Wong P.W., Lee S., Huttner A.J., Iwasaki A. (2018). Antiviral CD8 T cells induce Zika-virus-associated paralysis in mice. Nat. Microbiol..

